# ABA-dependent control of *GIGANTEA* signalling enables drought escape via up-regulation of *FLOWERING LOCUS T* in *Arabidopsis thaliana*

**DOI:** 10.1093/jxb/erw384

**Published:** 2016-10-12

**Authors:** Matteo Riboni, Alice Robustelli Test, Massimo Galbiati, Chiara Tonelli, Lucio Conti

**Affiliations:** Department of BioSciences, Università degli Studi di Milano, Via Celoria 26, 20133, Milan, Italy

**Keywords:** Abscisic acid (ABA), adaptation, drought stress, florigen expression, flowering, photoperiod.

## Abstract

One strategy deployed by plants to endure water scarcity is to accelerate the transition to flowering adaptively via the drought escape (DE) response. In *Arabidopsis thaliana*, activation of the DE response requires the photoperiodic response gene *GIGANTEA* (*GI*) and the florigen genes *FLOWERING LOCUS T* (*FT*) and *TWIN SISTER OF FT* (*TSF*). The phytohormone abscisic acid (ABA) is also required for the DE response, by promoting the transcriptional up-regulation of the florigen genes. The mode of interaction between ABA and the photoperiodic genes remains obscure. In this work we use a genetic approach to demonstrate that ABA modulates GI signalling and consequently its ability to activate the florigen genes. We also reveal that the ABA-dependent activation of *FT*, but not *TSF*, requires *CONSTANS* (*CO*) and that impairing ABA signalling dramatically reduces the expression of florigen genes with little effect on the *CO* transcript profile. ABA signalling thus has an impact on the core genes of photoperiodic signalling *GI* and *CO* by modulating their downstream function and/or activities rather than their transcript accumulation. In addition, we show that as well as promoting flowering, ABA simultaneously represses flowering, independent of the florigen genes. Genetic analysis indicates that the target of the repressive function of ABA is the flowering-promoting gene *SUPPRESSOR OF OVEREXPRESSION OF CONSTANS1* (*SOC1*), a transcription factor integrating floral cues in the shoot meristem. Our study suggests that variations in ABA signalling provide different developmental information that allows plants to co-ordinate the onset of the reproductive phase according to the available water resources.

## Highlight

We reveal that ABA affects flowering through two independent regulatory mechanisms: the activation of GI and CO functions upstream of the florigen genes and the down-regulation of *SOC1* signalling.

## Introduction

Water deprivation triggers several physiological adjustments at the cellular and organ levels (Shinozaki and Yamaguchi-Shinozaki, 2007). Depending on the intensity and duration of drought episodes, some plants can also respond adaptively, by activating the drought escape (DE) response (Franks, 2011; Riboni *et al.*, 2013, 2014; Kazan and Lyons, 2016). DE allows plants to accelerate the floral transition and set seeds before drought conditions become too severe. While escaping the potentially lethal effects of drought, plants undergoing DE usually produce fewer fruits and seeds, indicating a trade-off between plant survival and successful seed set (Su *et al.*, 2013; Kenney *et al.*, 2014). Therefore, a more precise understanding of the mechanisms leading to DE is of fundamental importance to assess the diverse modes of adaptations of natural plant populations as well as to produce crops with increased productivity under water deprivation (Lovell *et al.*, 2013; Kooyers, 2015).

*Arabidopsis thaliana* is a facultative long-day (LD) plant, flowering much earlier under LDs, typical of spring/summer compared with short days (SDs). The DE response occurs under LDs, but not SDs, indicating an interdependence between DE and photoperiod signalling in Arabidopsis (Han *et al.*, 2013; Riboni *et al.*, 2013). The photoperiodic pathway comprises three key genes, whose regulation and activity are required for the correct interpretation of day length: *GIGANTEA* (*GI*), *CONSTANS* (*CO*), and *FLOWERING LOCUS T* (*FT*) (Putterill *et al.*, 1995; Fowler *et al.*, 1999; Kardailsky *et al.*, 1999; Kobayashi *et al.*, 1999; Park *et al.*, 1999). *CO* encodes a nuclear protein (Putterill *et al.*, 1995; Samach *et al.*, 2000) able to induce the transcriptional activation of the florigen genes *FT* and *TWIN SISTER OF FT* (*TSF*) (An *et al.*, 2004; Yamaguchi *et al.*, 2005; Jang *et al.*, 2009). Accumulation of the *CO* transcript during the day depends on LIGHT OXYGEN VOLTAGE (LOV) domain-containing, blue light receptor FLAVIN-BINDING, KELCH REPEAT F-BOX 1 (FKF1), and GI (Imaizumi *et al.*, 2003, 2005; Sawa *et al.*, 2007; Fornara *et al.*, 2009; Song *et al.*, 2012). Formation of a GI–FKF1 complex is stimulated by blue light and leads to degradation of the *CO* transcriptional repressors CYCLING DOF FACTORs (CDFs) (Imaizumi *et al.*, 2005; Fornara *et al.*, 2009), allowing *CO* transcription. While *CO* transcript accumulation broadly occurs under both LDs and SDs, CO protein is activated to promote flowering only under LDs when *CO* mRNA peaks in the light phase at the end of the day (Suarez-Lopez *et al.*, 2001). Such a daily pattern of CO protein accumulation is controlled by several types of photoreceptors, which generate a peak of CO abundance in coincidence with dusk under LDs (Valverde *et al.*, 2004; Jang *et al.*, 2008; Liu *et al.*, 2008; Zuo *et al.*, 2011; Lazaro *et al.*, 2012; Song *et al.*, 2012).

CO promotes *FT* transcription in the phloem companion cells (Adrian *et al.*, 2010). However, FT protein acts as a florigenic signal, moving long distance to the shoot apical meristem (SAM), where it interacts with the bZIP transcription factors FLOWERING LOCUS D (FD) and FD PARALOGUE (FDP) to orchestrate the floral transition (Abe *et al.*, 2005; Wigge *et al.*, 2005; Corbesier *et al.*, 2007; Jaeger and Wigge, 2007; Mathieu *et al.*, 2007; Jaeger *et al.*, 2013). Amongst the early targets of the FT–FD complex is *SUPPRESSOR OF OVEREXPRESSION OF CONSTANS 1* (*SOC1*), a MADS box transcription factor, which integrates several floral pathways in the SAM (Borner *et al.*, 2000; Lee *et al.*, 2000; Samach *et al.*, 2000; Moon *et al.*, 2003; Searle *et al.*, 2006; Jang *et al.*, 2009; Wang *et al.*, 2009; Lee and Lee, 2010).

Besides photoperiod, *FT* activation is modulated by several environmental cues (Pin and Nilsson, 2012), including drought stress (Riboni *et al.*, 2013). The activation of *FT* by drought requires abscisic acid (ABA), a key hormone mediating water stress stimuli (Riboni *et al.*, 2013). ABA derives from the carotenoid zeaxanthin synthetized in chloroplasts. Here, different enzymes, including ABA1, transform zeaxanthin into xanthoxin prior to its translocation to the cytoplasm where another set of enzymes, namely ABA2, complete the last biosynthetic steps leading to bioactive ABA (Nambara and Marion-Poll, 2005). Three signalling proteins form the core ABA signalling, including the PYRABACTIN RESISTANCE (PYR)/REGULATORY COMPONENT OF ABA RECEPTOR (RCAR), the PROTEIN PHOSPHATASE 2Cs (PP2Cs), and SNF1-RELATED PROTEIN KINASE 2s (SnRK2s) (Cutler *et al.*, 2010). The PYR/RCARs are the ABA receptors, the PP2Cs [e.g. the *ABA INSENSITIVE 1* (*ABI1*) gene] act as negative regulators of the pathway, and the SnRK2s act as positive regulators of downstream signalling (Ma *et al.*, 2009; Park *et al.*, 2009).

ABA-deficient mutants *aba1* and *aba2* display a general delay in flowering in LDs, which is more evident under drought conditions as well as reduced florigen transcript accumulation. Similar to *aba1*, mutants of *GI* are impaired in DE, and display no florigen up-regulation under drought conditions (Riboni *et al.*, 2013). The nature of GI signalling upstream of the florigen activation during DE is however unclear. Because no DE occurs in wild-type plants under SDs, one can conclude that GI activates DE by mediating photoperiodic signals. However, such a mechanism does not appear to require CO activity, since *co* mutants display a normal DE response (Han *et al.*, 2013; Riboni *et al.*, 2013). Modes of GI-dependent but CO-independent pathways include the activation of a class of miRNA, the *miR172*, which targets the *APETALA 2*-like factors that repress *FT* and other flowering genes (Jung *et al.*, 2007; Mathieu *et al.*, 2009). The role of GI in DE may also be indirect and/or biochemically distinct from its role in photoperiodic flowering. For example, GI affects phytochrome signalling (Huq *et al.*, 2000; Martin-Tryon *et al.*, 2007; Oliverio *et al.*, 2007), clock function (Park *et al.*, 1999; Fowler *et al.*, 1999; Mizoguchi *et al.*, 2005), and several plant–environment responses, namely salinity and freezing tolerance (Han *et al.*, 2013; Kim *et al.*, 2013*b*; Fornara *et al.*, 2015; Xie *et al.*, 2015), through mechanisms which cannot be fully ascribed to the canonical photoperiodic signalling cascade.

In this study, tests were carried out to elucidate the role of GI signalling in the DE response. We analysed the DE response and patterns of florigen accumulation in Arabidopsis mutant backgrounds with varying levels of *CO* and in the presence or absence of GI. To assess the role of ABA in the GI-mediated pathway, we combined mutants impaired in ABA signalling with transgenic plants overexpressing *GI*. We show that impaired ABA signalling affects GI downstream functions and/or activity, thus causing reduced accumulation of florigen genes, but no effects on *CO* accumulation. Our results also clarify the relationship between GI and CO in the context of DE response by showing that the drought/ABA-dependent activation of *FT* requires *CO*. In contrast, up-regulation of *TSF* under drought stress can occur without CO, thus expanding the repertoire of regulatory mechanisms of florigen gene activation in plants. Alongside these results, we also demonstrate a florigen-independent floral repressive role for ABA in flowering, which requires *SOC1*. The transition to flowering under drought conditions thus depends on activation of separate ABA-dependent developmental programmes.

## Materials and methods

### Plant materials and growing conditions

In this study, we used wild-type *Arabidopsis thaliana* plants, ecotype Columbia (Col-0) or Landsberg *erecta* (L*er*). Mutant or transgenic lines were obtained from the Nottingham Arabidopsis Stock Centre or other laboratories as detailed in Supplementary Table S3 at *JXB* online. Seeds were stratified in the dark at 4 °C for 2 d before sowing, and plants grown in a controlled-environment cabinet at a temperature of 18–23 °C, 65% relative humidity, under either LD (16 h light/8 h dark) or SD (8 h light/16 h dark) photoperiods. Light was provided by cool white fluorescent tubes (Philips Lighting, F36W/33-640 36W) at a fluence of 120–150 μmol m^−2^ s^−1^ (photosynthetically active radiation). The procedures to impose drought stress, and perform photoperiod shift experiments were previously detailed (Riboni *et al.*, 2013).

**Fig. 1.**
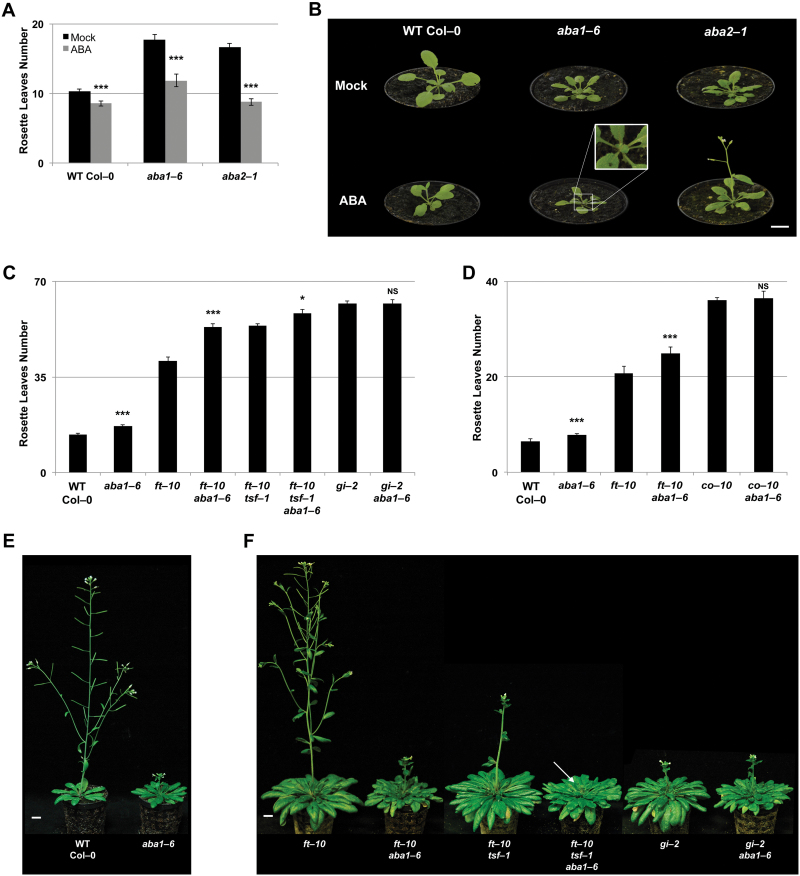
ABA activates flowering through GI, CO, and the florigen genes. (A) Mean number of rosette leaves of the wild type (Col-0) and ABA-deficient mutant plants grown under LDs and treated with ABA or mock treated. Error bars represent ±SE, *n*=15. Student’s *t*-test *P*-values ≤0.001 (***) compared with mock treatment. (B) Images of representative 24-day-old plants of the indicated genotypes grown under LDs and treated with ABA or mock treated. Inset of *aba1-6* shows a visible inflorescence. (C and D) Mean number of rosette leaves of the wild type (Col-0) and flowering time mutants grown under LDs. Error bars represent ±SE, *n*=15. Student’s *t*-test *P*-values ≤0.05 (*), ≤0.001 (***), >0.05 not significant (NS) are shown to indicate differences between mutants and the corresponding mutant containing the *aba1-6* allele. The experiment in (D) was performed under semi-controlled greenhouse conditions. (E) and (F), Images of representative plants of the indicated genotypes grown under LDs. (E) Wild-type Col-0 and *aba1-6* mutant plants are 4 weeks old, (F) *ft-10*, *ft-10 aba1-6*, *ft-10 tsf-1*, *ft-10 tsf-1 aba1-6*, *gi-2*, and *gi-2 aba1-6* mutant plants are 14 weeks old. The arrow indicates the visible bolt in *ft-10 tsf-1 aba1-6.* Scale bars=1 cm. (This figure is available in colour at *JXB* online.)

Experiments in Fig. 1B were performed in a greenhouse, with a semi-controlled climate. Temperature was 19–23 °C and relative humidity was set at 65%. Natural light was supplemented by incandescent (metal halide) lamps when external light was <150 μmol m^−2^ s^−1^ (photosynthetically active radiation) in an LD photo cycle. Two independent greenhouse experiments were performed (autumn 2015 in Milan). ABA application experiments were performed by daily supplying 2 ml of ABA (25 μM) or mock solutions (0.025% v/v ethanol) 7 h after dawn. ABA applications started 3 d after germination and continued for 21 d. Each Arabasket pot was fitted with a pipette tip to facilitate the application of the solutions directly in the soil and thus in contact with roots (Supplementary Fig. S1).

### Isolation of double mutants and genotyping

Mutant combinations were generated by crossing. The *aba1-6* mutation was genotyped as described in Riboni *et al.* (2013). *ft-10* mutants were selected on Murashige and Skoog plates containing Sulafadiazide as described (Rosso *et al.*, 2003). *abi1-1* mutants were selected by genomic PCR amplification with primers flanking the *abi1-1* polymorphism followed by digestion with *Nco*I. Genotyping primers for *tsf1-1*, *co-10*, and *abi1-1* are listed in Supplementary Table S4. Plants carrying the *gi-2* and *soc1-1* alleles were selected based on their late flowering phenotype, while *elf3-1* mutants were selected on the basis of their early flowering and long hypocotyl.

### RNA extraction and real-time qPCR

Total RNA was extracted with TRIzol reagent (Invitrogen). A 1.5 µg aliquot of total RNA was used for cDNA synthesis with the SuperScript VILO cDNA Synthesis Kit (Invitrogen). Quantitative real-time PCR was performed as previously detailed (Riboni *et al.*, 2013) and PCR primers are provided in Supplementary Table S4.

### Molecular cloning and plant transformation

A 2.2 kbp promoter region upstream of the *ABI1* coding sequence was cloned using the Gateway cloning technology (Invitrogen) with specifics primers (Supplementary Table S4). The promoter was cloned into the pDONR207 entry vector (Invitrogen) and moved into the pBGWFS7 destination vector (Karimi *et al.*, 2002). The resulting plasmid was introduced into *Agrobacterium* strain GV3101 (pMP90RK) (Koncz and Schell, 1986) and transformed in wild-type Col-0 plants by floral dip (Clough and Bent, 1998). Six independent transgenic plants were selected based on the segregation of Basta resistance in a Mendellian 3:1 ratio in the T_2_ generation and analysed for β-glucuronidase (GUS) staining.

### GUS assay

Plants were grown under LDs and sampled at the indicated Zeitgeber time (ZT) time. Tissue was fixed for 30 min at 0 °C with 90% (v/v) acetone. After being washed in 50 mM sodium phosphate buffer, pH 7.0 they were incubated for 14 h at 37 °C in staining solution [0.5 mg ml^–1^ X-Gluc (5-bromo-4-chloro-3-indolyl-β-d-glucuronide), 50 mM sodium phosphate buffer, pH 7.0, 0.5 mM potassium ferrocyanide, 0.5 mM potassium ferricyanide, and 0.1% (v/v) Triton X-100]. Samples were cleared with a chloral hydrate:glycerol:water solution (8:1:2, v/v/v) for 3 h and then stored in 70% (v/v) ethanol before GUS histochemical reactions were visualized under a stereomicroscope.

## Results

### ABA promotes FT expression through CO

Mutants of *aba1-6* were later flowering compared with the wild type under LDs (Fig. 1A–C). We confirmed a similar late flowering phenotype in *aba2-1* mutants, defective in the final steps of ABA biosynthesis (Finkelstein, 2013). Soil applications of ABA could accelerate flowering in wild-type plants, reminiscent of DE response (Fig. 1A; Supplementary Table S1) (Koops *et al.*, 2011). Using this experimental set-up, we could also largely rescue the late flowering of *aba1-6* and *aba2-1* mutants, indicating a role for ABA as an activator of flowering (Fig. 1A, B).

We have previously demonstrated that ABA activates flowering under LDs but not SDs and that ABA affects photoperiodic signalling upstream of *FT* expression (Riboni *et al.*, 2013). To understand how ABA interacts with photoperiod signalling to affect flowering, we generated combinations of ABA-deficient (*aba1-6*) and photoperiodic pathway mutants (Fig. 1C, D; Supplementary Table S1). Consistent with lack of flowering defects of *aba1-6* under SDs (Riboni *et al.*, 2013), double mutants of *gi-2 aba1-6* displayed a similar flowering time compared with *gi-2* single mutants under LDs (Fig. 1C, F). Since double mutants of *ft-10 aba1-6* were later flowering than *ft-10* single mutants, ABA could affect flowering time via other florigen genes, namely *TSF* (Fig. 1C, F). The *tsf-1 ft-10 aba1-6* triple mutants were slightly later flowering than *tsf-1 ft-10* double mutants (Fig. 1C, F). *TSF* thus contributes to the late flowering phenotype of *ft-10 aba1-6* plants although ABA also appears to have an effect on other floral pathways, independent of *FT* and *TSF*. Interestingly, double mutants of *co-10 aba1-6* were similar to *co-10* single mutants, indicating that *CO* is also required for the late flowering phenotype of *aba1-6* mutants (Fig. 1D).

**Fig. 2.**
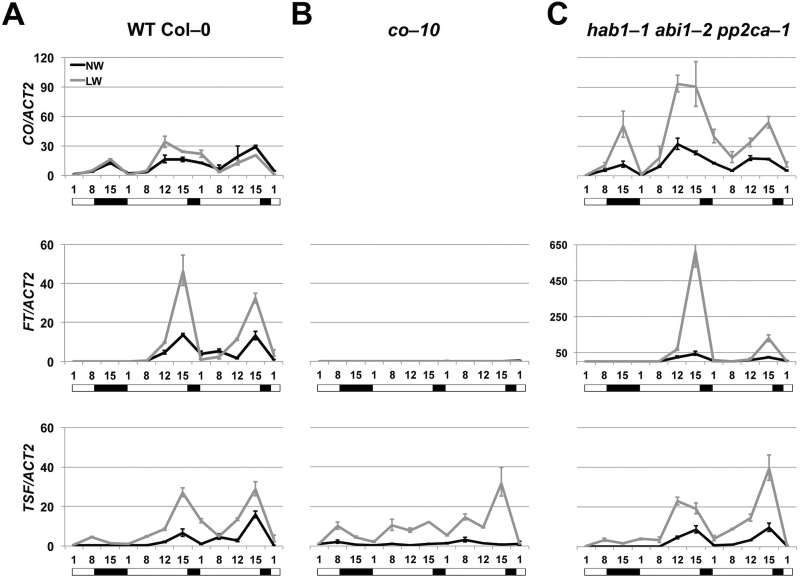
CO is required for the activation of FT under drought stress. (A–C) Real-time qPCR of *CO*, *FT*, and *TSF* transcripts in 3-week-old wild-type (Col-0) (A), *co-10*, (B) and *hab1-1 abi1-2 pp2ca-1* (C) seedlings. Plants were subject to normal watering (NW; black lines) or low watering (LW; grey lines) regimes and harvested at the indicated time points in coincidence with the light phase (open bar) or in the dark (black bar) during an SD to LD shift. At each time point, values represent fold change variations of *CO*, *FT*, and *TSF* transcript levels relative to Col-0 under NW. *ACT2* expression was used for normalization; error bars represent the SD of two technical replicates. A representative experiment of two biological replicates is shown.

Unlike *gi*, *co* mutants generate a DE response, indicating that high levels of ABA accumulation, as a result of drought stress, may eventually overcome CO function to activate flowering (Riboni *et al.*, 2013). To test whether drought could activate the florigen genes in the absence of a functional CO protein we grew wild-type and *co-10* mutant plants under control or water stress conditions in SDs before shifting to LDs to induce a photoperiodic response. As expected, in wild-type plants *FT* expression was strongly up-regulated during the photo-extension period and even further increased under low watering conditions (Fig. 2A). In the *co-10* mutants, the levels of *FT* transcripts were barely detectable at any time point, independent of the watering regime, indicating that drought stress cannot cause *FT* up-regulation in the absence of a functional *CO* (Fig. 2B). The pattern of accumulation of *TSF* showed diurnal oscillations similar to those of *FT* in wild-type plants, peaking at dusk during the photo-extension period (Fig. 2A, B). Similar to *FT*, *TSF* expression was increased in coincidence with the photo-extension period under drought conditions. Furthermore in *co-10* mutants, *TSF* levels were much lower compared with the wild type under normal watering conditions, confirming a role for *CO* in *TSF* transcriptional activation (Yamaguchi *et al.*, 2005; Jang *et al.*, 2009). Surprisingly, drought stress caused *TSF* up-regulation in the *co-10* background, partially resuming its diurnal cycle with peaks at ZT8 under the SD part of the experiment and at ZT15 following a photo-extension. Slightly increased *TSF* levels were observed during SDs under drought conditions (on average 3.8 ± 1.6-fold) but this was not correlated with a DE phenotype under SDs in *co-10* mutants (Fig. 2B, D). Thus, unlike *FT*, *TSF* can be up-regulated under drought conditions in a *CO*-independent manner.

### GI is required for DE downstream of CO transcriptional activation

**Fig. 3.**
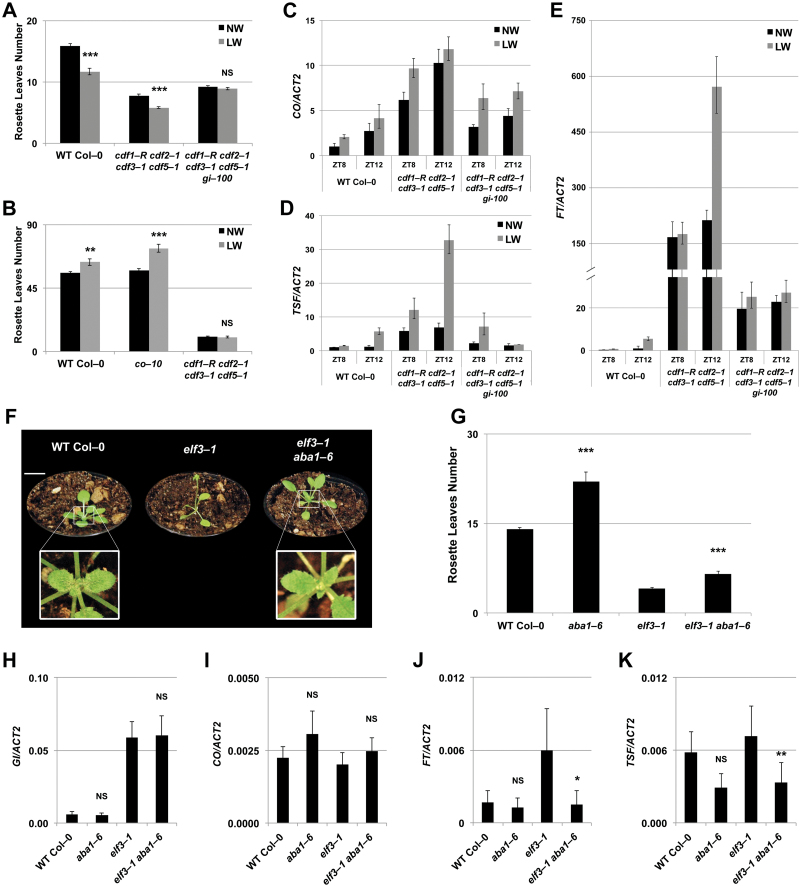
ABA promotes GI and CO functions to activate the florigen genes. (A and B) Mean number of rosette leaves of the wild type (Col-0) and flowering time mutants subject to normal watering (NW; black bars) or low watering (LW; grey bars) regimes grown under LDs (A) and SDs (B). Error bars represent ±SE *n*=15. Student’s t-test *P* values ≤0.001 (***), >0.05 not significant (NS) compared with NW. (C–E) Real-time qPCR of *CO* (C), *TSF* (D), and *FT* (E) transcripts in 2-week-old wild-type (Col-0), *cdf1-R cdf2-1 cdf3-1 cdf5-1*, and *cdf1-R cdf2-1 cdf3-1 cdf5-1 gi-100* seedlings. Plants were subject to NW (black columns) or LW (grey columns) regimes and harvested at the indicated Zeitgeber time during a shift from SDs to LDs. ZT8 represents dusk in SDs and ZT12 represents 4 h of photo-extension. At each time point, values represent fold change variations of *CO*, *FT*, and *TSF* transcript levels relative to the wild type at ZT8 under NW. *ACT2* expression was used for normalization; error bars represent the SD of two technical replicates. A representative experiment of two biological replicates is shown. (F) Images of representative plants grown under LDs for 27 d. Insets shows a visible inflorescence in *elf3-1 aba1-6* double mutants, which is not visible in the wild type. (G) Mean numbers of rosette leaves of the wild type (Col-0) and mutants under LDs. Error bars represent ±SE, *n*=5–12. Student’s *t*-test *P*-values ≤0.001 (***) are shown to indicate differences between mutants and the corresponding mutant containing the *aba1-6* allele. (H–K) Real-time qPCR of *GI* (H), *CO* (I), *FT* (J), and *TSF* (K) transcripts in 12-day-old seedlings grown under LDs and sampled at ZT16. Data shown are from 5–6 biological replicates. Error bars represent ±SD. Differences between the wild type versus *aba1-6* and *elf3-1* versus *elf3-1 aba1-6* double mutants are here highlighted with *P*-values ≤0.01 (**), ≤0.05 (*), >0.05 not significant (NS), one-way ANOVA with Tukey’s HSD (honestly significant difference) test. (This figure is available in colour at *JXB* online.)

Our experiments indicate that ABA promotes *FT* transcript accumulation through *CO*. However, *CO* transcript levels are not greatly affected by drought stress or when ABA level are reduced (Han *et al.*, 2013; Riboni *et al.*, 2014). Here we wanted to test whether drought could affect flowering downstream of *CO* transcriptional activation events, by analysing mutants of *cdf1-R cdf2-1 cdf3-1 cdf5-1*, hereafter referred to as *cdf1235*, characterized by constitutively elevated *CO* levels (Fornara *et al.*, 2009). The *cdf1235* mutants flowered early and produced a DE response quantitatively similar to that of the wild type under LDs (Fig. 3A). Despite their early flowering phenotype under SDs, *cdf1235* plants did not produce any DE response (Fig. 3B), suggesting a requirement for LDs in DE response, even when *CO* levels are elevated (Fornara *et al.*, 2009) (Fig. 3C). We therefore compared the flowering time and DE response of the quadruple *cdf1235* mutant with that of *gi cdf1235* quintuple mutants under LDs (Fig. 3A). As previously shown, mutants of *cdf1235* are slightly earlier flowering than *gi cdf1235* under normal watering conditions (Fornara *et al.*, 2009). However, while the *cdf1235* mutants produced a DE response, the *gi cdf1235* did not (Fig. 3A). We next sought to ascertain if the lack of DE response in the *gi cdf1235* mutants was correlated with impaired transcriptional up-regulation of the florigen genes under drought stress. Control and water-stressed wild-type, *cdf1235* and *gi cdf1235* plants were grown under SD conditions for 2 weeks before shifting to LDs, and transcript levels were analysed at ZT8 (corresponding to dusk in the SDs) and ZT12 (4 h after the photo-extension) (Fig. 3C–E). As expected, the levels of *CO* transcript were generally higher in *cdf1235* and *gi cdf1235* mutants as compared with the wild type. Under drought conditions, we observed a small increase in *CO* transcript abundance in all the genotypes analysed at any time point, suggesting a contribution of drought stress in *CO* transcript accumulation (Fig. 3C). We finally determined how different patterns of *CO* transcript were correlated with accumulation of florigen genes (Fig. 3D, E). Under well-watered conditions, mutants of *cdf1235* showed the largest *FT* and *TSF* transcript accumulations before and after the photo-extension period. Mutants of *gi cdf1235* displayed levels of *FT* and *TSF* intermediate between the wild type and the *cdf1235* mutants. This is probably as a result of residual CDF-mediated repression in *cdf1235* on both *CO* and *FT* promoters (Fornara *et al.*, 2009; Song *et al.*, 2012). However, while both the wild type and the *cdf1235* mutants showed a significant and similar up-regulation of *FT* and *TSF* under drought stress conditions at ZT12 (2- to 4-fold, respectively), no such up-regulation occurred in the *gi cdf1235* mutants (Fig. 3D, E).

In a complementary approach, we asked whether ABA production might be required for *FT* transcriptional activation when *GI* levels are increased. Mutants of *early flowering 3* (*elf3*) are extremely early flowering, accumulate high levels of *FT*, and present increased accumulation of *GI* transcript and GI protein (Fowler *et al.*, 1999; Kim *et al.*, 2005; Yu *et al.*, 2008). This early flowering phenotype requires ABA since *elf3-1 aba1-6* double mutants were significantly later flowering compared with *elf3-1* single mutants (Fig. 3F, G). *FT* and *TSF* transcript levels were slightly but not significantly reduced in *aba1-6* mutants compared with the wild type at this early developmental stage (Fig. 3J, K; Supplementary Table S2). However, double mutants of *elf3-1 aba1-6* had a significant reduction in both *FT* and *TSF* levels compared with the *elf3-1* single mutants (Fig. 3J, K; Supplementary Table S2). The reduced levels of *FT* and *TSF* in *elf3-1 aba1-6* compared with *elf3-1* mutants were not caused by diminished *GI* or *CO* transcript accumulations (Fig. 3H, I; Supplementary Table S2), indicating that ABA might be required for the activation of GI and CO signalling.

### ABA signalling genes control FT transcript accumulation with little effect on CO

We analysed ABA-hypersensitive mutants plants *hab1-1 abi1-2 pp2ca-1*, impaired in three ABA-related PP2C phosphate genes, under different watering and photoperiodic conditions (Rubio *et al.*, 2009). Consistent with previous observations, mutants of *hab1-1 abi1-2 pp2ca-1* had much increased (up to 6-fold) levels of *FT* compared with the wild type under LDs (Riboni *et al.*, 2013) (Fig. 2C). The experiment in Fig. 2C also shows that *FT* expression was even further activated under drought conditions compared with the wild type (up to 13.3-fold). In contrast, *TSF* expression was not clearly increased in *hab1-1 abi1-2 pp2ca-1* plants compared with the wild type under any watering condition. No *FT* or *TSF* up-regulation occurred under SDs in the *hab1-1 abi1-2 pp2ca-1* mutants under any watering condition.

Under control conditions the strong up-regulation of *FT* in *hab1-1 abi1-2 pp2ca-1* plants was not caused by increased *CO* levels, which were comparable with those observed in the wild type (Fig. 2C). Increased levels of *CO* were, however, observed in the *hab1-1 abi1-2 pp2ca-1* mutants under drought stress, indicating that high levels of ABA signalling can ultimately induce the transcriptional activation of *CO* (Koops *et al.*, 2011; Yoshida *et al.*, 2014).

**Fig. 4.**
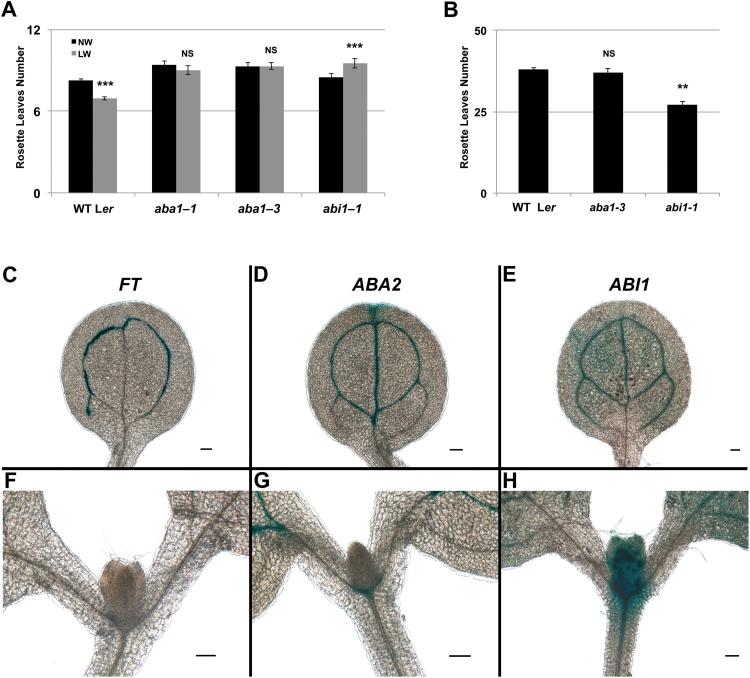
A negative role for ABA in flowering. (A and B) Mean number of rosette leaves of the wild type (L*er*) and ABA-deficient or signalling mutants grown under LDs and subject to normal watering (NW; black bars) or low watering (LW; grey bars) regimes (A), or under SDs in NW regime (B). Error bars represent ±SE *n*=15. Student’s *t*-test *P*-values ≤0.001 (***), >0.05 not significant (NS), compared with NW (A). One-way ANOVA with Tukey’s HSD (honestly significant difference) test *P*-values ≤0.01 (**), >0.05 not significant (NS), compared with the wild type (B). (C–H) Histochemical GUS detection in transgenic seedlings expressing *pFT::GUS* (C) and (F), *pABA2::GUS* (D) and (G), and *pABI1::GUS* (line # 1) (E) and (H) in the Col-0 background, scale bars=100 µm. (This figure is available in colour at *JXB* online.)

To explore further the role of ABA signalling in the transcriptional control of *FT*, we analysed *abi1-1* mutant plants (L*er* background), carrying a dominant mutation in the *PP2C* phosphatase *ABI1* (Koornneef *et al.*, 1984) which results in severely reduced ABA responses. *abi1-1* mutant plants did not show flowering defects under LDs, but exhibited an early flowering phenotype under SDs, consistent with previous observations (Martínez-Zapater *et al.*, 1994; Chandler *et al.*, 2000) (Fig. 4A, B). Ruling out an ecotype-specific effect for ABA action in flowering, the ABA biosynthetic mutants *aba1-1* and *aba1-3* (L*er* background) showed a marginal late flowering phenotype compared with the wild type under LDs (ANOVA *P*<0.01 and *P*<0.05, respectively), but no defects under SDs (Fig. 4A, B). The late flowering phenotype of these *aba1* mutants was more pronounced under drought conditions and LDs, indicative of a reduced DE response (Fig. 4A). Mutants of *abi1-1* were even more impaired in the DE response compared with the *aba1* alleles, producing on average 14 ± 2% more leaves (*n* = 8 independent experiments, 15 plants each), relative to the untreated control.

**Fig. 5.**
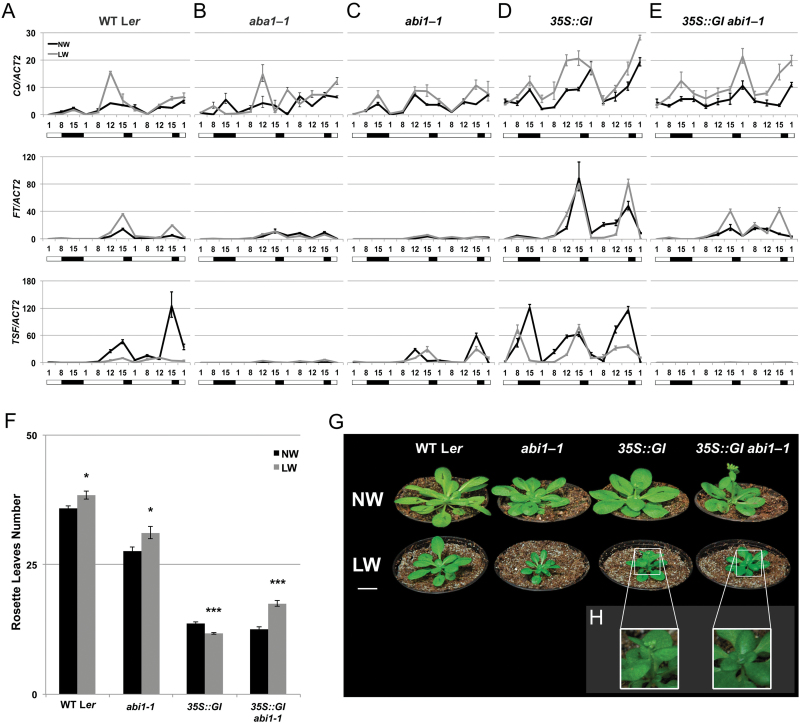
ABA activates GI signalling and florigen expression with little effect on CO transcript accumulation. (A–E) Real-time qPCR of *CO*, *FT*, and *TSF* transcripts in 2-week-old wild-type (L*er*) (A), *aba1-1* (B), *abi1-1* (C), *35S*::*GI* (D), and *35S*::*GI abi1-1* (E) seedlings. Plants were subject to normal watering (NW; black lines) or low watering (LW; grey lines) regimes and harvested at the indicated time points in coincidence with the light phase (open bar) or in the dark (black bar) during an SD to LD shift. At each time point, values represent fold change variations of *CO*, *FT*, and *TSF* transcript levels relative to L*er* under NW. *ACT2* expression was used for normalization; error bars represent the SD of two technical replicates. A representative experiment of two biological replicates is shown. (F) Mean number of rosette leaves of the wild type (L*er*) and mutants grown under SDs and subject to NW (black bars) or LW (grey bars) regimes, Error bars represent ±SE *n*=15. Student’s *t*-test *P*-values ≤0.05 (*), ≤0.001 (***) compared with NW. (G) Images of representative 5-week-old plants of the indicated genotypes grown under SDs and subject to NW or LW regimes. Scale bar=1 cm. (H) Higher magnification of LW *35S*::*GI* and *35S*::*GI abi1-1* plants shown in (G). Note the appearance of a bolt in *35S*::*GI* but not in *35S*::*GI abi1-1*.

We next analysed the pattern of accumulation of the florigen genes in *abi1-1* plants. As expected, in wild-type plants, the accumulation of *FT* was strongly induced under drought conditions in a photoperiod-dependent manner (Fig. 5A). *TSF* expression was instead down-regulated under drought conditions in the L*er* background, revealing an ecotype-specific effect for *TSF* regulation under drought (Fig. 5A). Lower levels of *FT* and *TSF* were observed in the *aba1-1* mutants compared with the wild type under both normal watering (*TSF*) and drought conditions (*FT* and *TSF*), confirming the contribution of ABA in both *FT* and *TSF* regulation (Fig. 5B) (Riboni *et al.*, 2013). Strikingly, in *abi1-1* plants the levels of *FT* were dramatically reduced compared not only with the wild type but also with *aba1-1* plants, under any watering condition analysed (Fig. 5C). Such low expression of the florigen genes did not depend on reduced *CO* transcript accumulation in *abi1-1* which was, if anything, up-regulated (Fig. 5C). Taken together, our data point to a model where ABA affects accumulation of florigen genes without an effect on *CO* expression.

Loss of PP2C function (as in *hab1-1 abi1-2 pp2ca-1*) results in increased *FT* transcript accumulation, while expression of a gain-of-function form of *ABI1* (as in *abi1-1*) leads to reduced *FT* activation. To determine whether the negative regulation of *ABI1* on *FT* expression could be exerted in the cells expressing *FT*, we fused a 2.2 kb promoter region of *ABI1* to the GUS reporter. We detected GUS staining in several independent transgenic T_2_ plants (*n* = 6) with comparable results, at ZT8, where *ABI1* transcript accumulation is highly abundant according to a publicly available data set (http://diurnal.mocklerlab.org; Mockler *et al.*, 2007). For comparison, we also studied the pattern of GUS activity in Arabidopsis transgenic lines marking the *FT* expression domain; the *ABA2* (Lin *et al.*, 2006; Kuromori *et al.*, 2014) and the *FT* promoter itself (Notaguchi *et al.*, 2008). Histochemical detection in young seedlings revealed that *ABI1* expression (Fig. 4E) occurred in the vasculature of cotyledons in a pattern similar to *ABA2* and *FT* (Fig. 4C, D), demonstrating an overlap between ABA biosynthesis and signalling genes in the tissue known to be the source of FT protein production. Broadly distributed GUS staining was also observed in the apical region of *ABI1::GUS* transgenic plants (Fig. 4H). This pattern of expression may also indicate a role for ABA signalling in the shoot apex.

### Impaired ABA signalling negatively affects GI action

Whether impairing ABA signalling affects GI action was tested by generating *abi1-1 35S::GI* plants. As previously observed, *35S::GI* plants had increased levels of *FT* under both SDs and LDs compared with the wild type (Mizoguchi *et al.*, 2005). Under drought conditions, *FT* expression was generally less responsive to drought in the *35S::GI* background compared with the wild type (Fig. 5D). The levels of *TSF* were much more increased in *35S::GI* plants compared with the wild type during the SD part of the experiment. However, no further up-regulation of *TSF* occurred as a result of drought stress compared with normal watering (Fig. 5D). The overaccumulation of *FT* observed in *35S::GI* plants was strongly rescued in the *abi1-1 35S::GI* mutants under any watering conditions (Fig. 5E). The levels of *TSF* transcript fell even more severely in *abi1-1 35S::GI* plants compared with *35S::GI*. Such reductions in florigen accumulation in *abi1-1 35S::GI* plants were not related to decreased *CO* levels as these were much higher than in the wild type (Fig. 5A, E). Interestingly the levels of *CO* in *abi1-1 35S::GI* plants were only mildly reduced compared with *35S::GI*, which could suggest that the negative role exerted by abi1-1 protein on GI signalling is more related to *FT* and *TSF* regulation rather than to *CO* (Fig. 5D, E).

Our data describe a regulatory role of ABA in GI signalling. Such ABA-mediated post-transcriptional activation of *GI* is consistent with previous observations on *35S::GI* plants showing a DE-responsive phenotype under SDs (Riboni *et al.*, 2013). In contrast, no DE response occurred in *abi1-1 35S::GI* mutants, which flowered much later compared with well-watered plants of the same genotype, although still earlier than *abi1-1* plants (Fig. 5F). Under normal watering conditions, double mutants of *abi1-1 35S::GI* had a similar flowering phenotype to *35S::GI* plants, despite showing reduced accumulation of the florigen genes (Fig. 5E, F). A similar observation could be made for *abi1-1* plants, which did not show flowering defects under LDs compared with the wild type, but had reduced florigen expression (Fig. 5A, C). We conclude that late flowering of *abi1-1* or *abi1-1 35S::GI* plants under drought stress cannot be solely ascribed to reduced florigen up-regulation.

### A negative role for ABA signalling in flowering

**Fig. 6.**
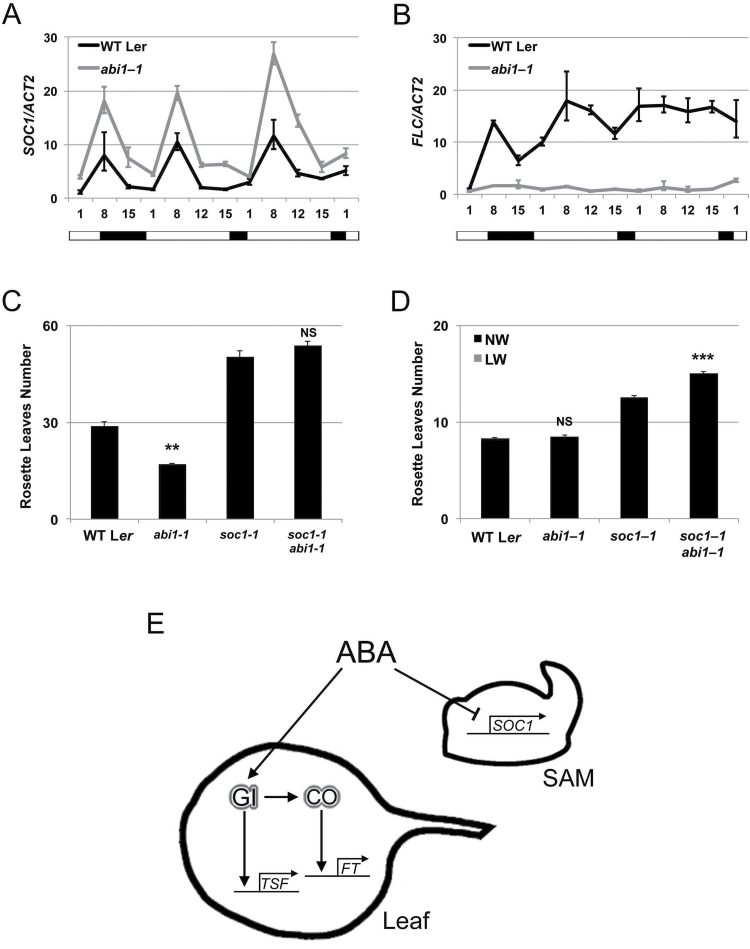
The inhibitory role of ABA in flowering requires SOC1. (A and B) Real-time qPCR of *SOC1* (A) and *FLC* (B) transcripts in 2-week-old wild-type (L*er*) and *abi1-1* seedlings. The experimental conditions were described in Fig. 5. *ACT2* expression was used for normalization; error bars represent the SD of two technical replicates. A representative experiment of two biological replicates is shown. (C and D) Mean number of rosette leaves of the wild type (L*er*) and mutants grown under SDs (C) or LDs (D). Error bars represent ±SE *n*=15. Differences between the wild type versus *abi1-1* and *soc1-1* versus *soc1-1 abi1-1* double mutants are here highlighted with *P*-values ≤0.001 (***), ≤0.01 (**), >0.05 not significant (NS), one-way ANOVA with Tukey’s HSD (honestly significant difference) test. (E) Model summarizing the proposed modes of ABA action in flowering. In the leaves, under LDs, drought promotes ABA accumulation leading to enhanced GI signalling and activation of florigen genes. CO protein is required for *FT* up-regulation, but not *TSF*. At the same time, at the shoot apex ABA represses flowering, downplaying *SOC1* signalling, independent of photoperiodic conditions.

The early flowering of *abi1-1* plants under SDs (Fig. 4B) implies that ABA signalling also exerts a negative role in flowering, which is usually undetectable under LDs or in ABA biosynthetic mutants (Fig. 4A). Supporting this model, we have previously reported a delay of flowering time under SDs in mutants of *hab1-1 abi1-2 pp2ca-1* and observed a similar phenotype also in *hab1-1 abi1-2 abi2-2* plants (Riboni *et al.*, 2013) (Supplementary Fig. S2). *abi1-1* mutants showed no increase in *FT* and *TSF* levels under SDs (Fig. 5B). In contrast, the accumulation of another floral integrator, *SOC1*, was increased in *abi1-1* plants as compared with the wild type under any photoperiodic condition (Fig. 6A). Mutants of *abi1-1* also had strongly reduced levels of *FLOWERING LOCUS C* (*FLC*) (Fig. 6B), a transcriptional repressor of *SOC1* which contributes to delaying flowering under drought condition (Riboni *et al.*, 2013; Y. Wang *et al.*, 2013; Shu *et al.*, 2016). Since *SOC1* integrates different floral pathways in the SAM (Moon *et al.*, 2003; Wang *et al.*, 2009; Song *et al.*, 2012, 2014) which promote flowering under SDs we created the *abi1-1 soc1-1* double mutants. Under SDs, these plants displayed a flowering time similar to the *soc1-1* single mutants. With respect to flowering time, *soc1-1* is thus completely epistatic to *abi1-1*, indicating that *SOC1* activity is required for the early flowering of *abi1-1* mutants under SDs (Fig. 6C).

Under LDs, *abi1-1 soc1-1* double mutants were later flowering than *soc1-1* single mutants (Fig. 6D). Thus, the knocking out of *SOC1* produces a novel flowering phenotype in the *abi1-1* background, consistent with ABA being able to affect flowering differentially in different domains of the plant; by promoting *FT* expression in the leaves and negatively regulating floral stimuli in the SAM (Fig. 6E).

## Discussion

A fundamental question related to the DE mechanism is how ABA signals are integrated in the photoperiodic flowering network. Here we provide evidence for how ABA controls *FT* gene expression under normal and drought stress conditions by affecting photoperiodic signalling. We also highlight a negative effect of ABA during the floral transition of Arabidopsis, which is independent of the photoperiodic pathway.

### ABA requires both GI and CO to regulate FT

Our genetic data point to a model where ABA requires both *GI* and *CO* to affect flowering under LDs through the transcriptional activation of the florigen genes. Since mutants of *ft-10 tsf-1 aba1-6* were still slightly later flowering than *ft-10 tsf-1*, it is possible that ABA may act on other pathways or through activation of *MFT*, a third florigen gene with a marginal role in flowering (Kim *et al.*, 2013*a*).

Expression and phenotypic analyses of *cdf1235*, *gi cdf1235*, as well as *aba1 elf3* mutants collectively suggest that ABA promotes GI and CO signalling upstream of the florigen genes. CO function is essential for the drought-dependent activation of *FT* (but not *TSF*) as demonstrated by the lack of *FT* accumulation in *co* mutants under drought conditions. Therefore, although we could not resolve the underlying molecular mechanism, our data underscore a regulatory role for ABA in stimulating photoperiodic signalling. In further support of this model, *35S::GI* plants under SDs generate a DE response, suggesting drought/ABA acting independently of *GI* transcript accumulation. Secondly, we observe a strong reduction in accumulation of florigen transcripts in *abi1-1 35S::GI* compared with *35S::GI* plants. Thirdly, the pattern of *CO* accumulation in *abi1-1* or *abi1-1 35S::GI* plants is unaltered compared with their respective controls, as opposed to the florigen levels, which are very low. In the light of our results, abi1-1 protein appears to affect a specific aspect of GI function (the activation of *FT*) without producing significant effects on the transcriptional profile of *CO* accumulation. Previous studies have demonstrated genetically separable roles for GI in regulating the circadian clock and flowering (Mizoguchi *et al.*, 2005; Martin-Tryon *et al.*, 2007) which could reflect distinct biochemical activities for GI in these two pathways. ABA might thus control a novel biochemical function of GI.

GI is found at different promoter locations of *FT* in association with transcriptional repressors including SHORT VEGETATIVE PHASE and TEMPRANILLO (Sawa and Kay, 2011). A substrate of the GI–FKF1 complex, CDF1, also binds to the *FT* promoter and acts as a transcriptional repressor (Sawa *et al.*, 2007). Furthermore, by activating *miR172* expression, *GI* directs post-transcriptional gene silencing of the *AP2*-type transcriptional repressors of *FT* (Jung *et al.*, 2007). Overexpression of a *miR172*-related miRNA of soybean facilitates the DE response, promotes *FT* accumulation under drought conditions, and increases ABA sensitivity of Arabidopsis (Li *et al.*, 2016). Thus, one role of GI could be to favour the recruitment of CO at the *FT* promoter by promoting the proteasome-dependent degradation or the post-transcriptional gene silencing of transcriptional repressors (such as *AP2-like*) in an ABA-dependent manner. Another, not mutually exclusive, model is that the combined presence of GI and ABA alters the pattern of CO protein accumulation during the day through an unknown mechanism. In addition to these post-transcriptional effects, there is evidence for other layers of transcriptional regulation of *CO* exerted by drought/ABA (Fig. 2C) (Koops *et al.*, 2011; Ito *et al.*, 2012; P. Wang *et al.*, 2013; Yoshida *et al.*, 2014). The contribution of these regulatory nodes to DE will require further studies. Regardless of the mechanisms involved and considering the role of the circadian clock in the control of ABA accumulation and response (Fukushima *et al.*, 2009), our results suggest that daily variations in ABA signalling may represent a further layer of regulation of CO protein function.

### Different modes of regulation of FT and TSF by drought

While *FT* and *TSF* share a common mechanism of transcriptional regulation through the photoperiodic pathway (Yamaguchi *et al.*, 2005; Jang *et al.*, 2009), they also display clear differences in their pattern of expression (Yamaguchi *et al.*, 2005), response to ambient temperature (Blázquez *et al.*, 2003), and other kinds of regulation (Michaels *et al.*, 2005; D’Aloia *et al.*, 2011; Liu *et al.*, 2014). In this work, we report variations in the transcriptional activations of *TSF* and *FT* in response to drought. Our expression studies on *co-10* mutants revealed that the expression of *TSF*, but not *FT*, is strongly induced by drought, even in the absence of functional CO. Previously we proposed a model whereby photoperiod-stimulated GI protein triggers a DE response via activation of the florigen genes, independent of *CO* (Riboni *et al.*, 2013). Based on our new results, this model only applies to *TSF* regulation, not *FT*. The DE response observed in the *co* mutants could therefore derive from residual *TSF* expression, which still depends on GI (Riboni *et al.*, 2013). Examples of *GI* acting independently of *CO* in activating the florigen genes have been described in the literature, but how these mechanisms are related to ABA signalling is unknown (Kim *et al.*, 2005; Mizoguchi *et al.*, 2005; Sawa and Kay, 2011). Other hormones modulate the expression of the florigen genes without an apparent contribution of *CO*. Cytokinin can induce the transcriptional activation of *TSF*, but not *FT*, irrespective of photoperiod conditions (D’Aloia *et al.*, 2011). Foliar applications of gibberellins under SDs promote flowering, at least in part through *FT* ad *TSF* and without a clear effect on *CO* transcript accumulation (Porri *et al.*, 2012). Similarly, there are examples of environmental cues activating *FT*, which do not fully require the activity of *CO* or *GI*, namely under elevated ambient temperature (Balasubramanian *et al.*, 2006). Here, we demonstrate that the activation of *TSF* can occur in the absence of *CO* under drought conditions but, unlike the previous examples, such activation requires *GI* (Riboni *et al.*, 2013).

### Multiple and contrasting roles of ABA in flowering

The role of ABA during the floral transitions is controversial, as both positive and negative effects of ABA have been reported (Domagalska *et al.*, 2010; Conti *et al.*, 2014). Depending on the site of application, ABA exerts opposite roles in flowering. Unlike leaf applications, we show that root applications of ABA promote flowering, consistent with previous data (Koops *et al.*, 2011). Also, this treatment largely rescues the late flowering of ABA biosynthetic mutants. In the light of these results, root applications fully mimic the positive role of endogenous ABA in flowering.

Impairing the function of ABA-activated kinases SnRK2.2/2.3/2.6 results in early flowering, especially under SDs, supporting a negative role for ABA in flowering (P. Wang *et al.*, 2013). Arguing against a direct negative role of the SnRK2s in the flowering network, overexpression of *SnRK2.6/OST1* causes a small flowering acceleration under LDs, not a delay (Zheng *et al.*, 2010). The negative role of ABA in flowering has been linked to the direct activation of *FLC* by ABA-stimulated bZIP transcriptional factor *ABSCISIC ACID-INSENSITIVE 5* (*ABI5*) and AP2/ERF domain-containing transcription factor *ABSCISIC ACID-INSENSITIVE 4* (*ABI4*) (Y. Wang *et al.*, 2013; Shu *et al.*, 2016). Such activation of *FLC* may account for the general reduction in *FT* transcript accumulation following exogenous ABA applications on leaves (Hoth *et al.*, 2002). The study of *abi1-1* plants under SDs supports this negative effect of ABA in flowering. ABA-deficient mutants do not produce similar flowering alterations under SDs, which could depend on ABA biosynthetic mutants still producing a sufficient amount of biologically active ABA (Léon-Kloosterziel *et al.*, 1996). The early flowering of *abi1-1* plants in SDs can be completely suppressed by mutations in *SOC1*, a floral integrator activating flowering in the SAM (Searle *et al.*, 2006). Elevated levels of *SOC1* transcript in *abi1-1* mutants also suggest a negative role for ABA in *SOC1* expression, perhaps mediated by *FLC* (Fig. 6A, B). The proposed positive role of ABA-activated ABI5 on *FLC* transcriptional activation is consistent with this model (Y. Wang *et al.*, 2013).

*abi1-1* plants do not present obvious flowering phenotypes under LDs despite impaired photoperiod-dependent accumulation of *FT*. We thus propose that the *abi1-1* mutants compensate for their defects in *FT* up-regulation with increased *SOC1* signalling. The late flowering phenotype of *abi1-1 soc1-1* compared with *soc1-1* under LDs is consistent with ABA playing antagonistic and spatially distinct roles in flowering, through the transcriptional activation of the florigen genes in the leaves and the repression of *SOC1* action in the shoot.

In addition to the ABA-dependent negative regulation of flowering, an ABA-independent floral repression mechanism emerged from the study of *abi1-1* plants. Under LDs, mutants of *abi1-1* exhibit a late flowering phenotype under drought stress, which is even more severe than *aba1* plants. We observed an even more pronounced delay in flowering under SDs in *abi1-1 35S::GI* plants upon drought stress compared with *35S::GI*. We interpret these results to indicate that the defects in florigen up-regulation of *abi1-1* contribute to the late flowering of *abi1-1* under drought stress. However, the levels of florigen expression in *abi1-1* were generally also low under normal watering conditions. Therefore, we hypothesize a further layer of negative regulation of flowering, which is triggered by drought stress and is probably independent of ABA (as it occurs in *abi1-1* plants). Both flowering-repressive mechanisms, the ABA-dependent and the ABA-independent mechanism, can be largely overcome under LDs, upon migration of the florigen protein in the SAM.

In conclusion, Arabidopsis plants have independent and contrasting mechanisms to modulate flowering according to water inputs; ABA stimulates GI and CO signalling to boost *FT* activation. Under drought conditions *TSF* activation is independent of *CO* and requires photoactivated GI. Simultaneously, ABA negatively regulates flowering through a pathway that requires *SOC1* (Fig. 6E), perhaps in conjunction with an ABA-independent type of regulation. Integration of these pathways in the SAM may provide plants with a flexible control of reproductive development under water stress and maximization of reproductive success.

## Supplementary data

Supplementary data are available at *JXB* online.

Figure S1. Method used for root applications of ABA.

Figure S2. Activated ABA signalling inhibits flowering under SDs.

Table S1. Flowering time of mutant and transgenic plants used in this study.

Table S2. Expression analysis of *aba elf3* mutant plants.

Table S3. Genotypes used in this study and references.

Table S4. Primers used in this study.

Supplementary Data

## Abbreviations:

DE: drought escape
LD: long day
SD: short day.
