# Supplementary material for: ABA-dependent control of GIGANTEA signalling enables drought escape via up-regulation of FLOWERING LOCUS T in Arabidopsis thaliana
Source: J Exp Bot. 2016 Oct 12;67(22):6309–22. doi: 10.1093/jxb/erw384 (PMC5181575; doi:10.1093/jxb/erw384)
Supplement: Supplementary Data [file supp_67_22_6309__index.html]

ABA-dependent control of GIGANTEA signalling enables drought escape via up-regulation of FLOWERING LOCUS T in Arabidopsis thaliana — ABA-dependent control of GIGANTEA signalling enables drought escape via up-regulation of FLOWERING LOCUS T in Arabidopsis thaliana — Supplementary Data 

# ABA-dependent control of *GIGANTEA* signalling enables drought escape via up-regulation of *FLOWERING LOCUS T* in *Arabidopsis thaliana*

## Supplementary Data

Data files

- Supplementary\_figures\_S1\_S2\_tables\_S1\_S4.pdf - Supplementary Data
