# Supplementary material for: ABA-dependent control of GIGANTEA signalling enables drought escape via up-regulation of FLOWERING LOCUS T in Arabidopsis thaliana
Source: J Exp Bot. 2016 Oct 12;67(22):6309–22. doi: 10.1093/jxb/erw384 (PMC5181575; doi:10.1093/jxb/erw384)
Supplement: Supplementary Data [file supp_erw384_Supplementary_figures_S1_S2_tables_S1_S4.pdf]

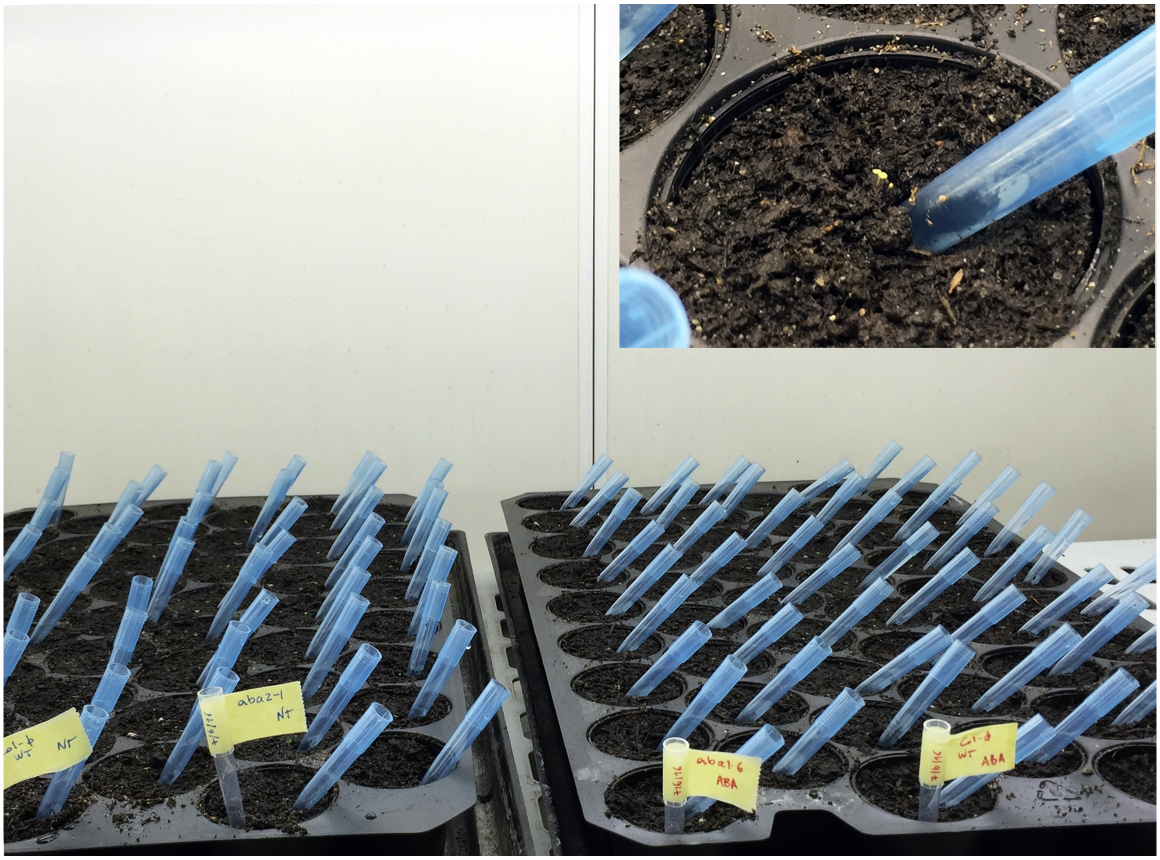

Figure S1

Method used for exogenous application of ABA. The ABA or mock solutions are applied in soil using a pipette tip as a funnel. Treatments started early in development (3 days post germination), thus before the floral transition.

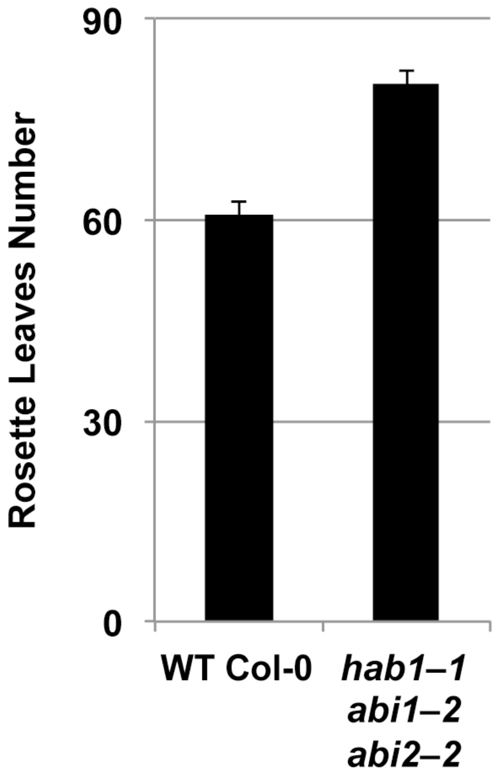

Figure S2

Activated ABA signaling inhibits flowering under SDs. Rosette leaves mean number at flowering of wild type (Col–0) and mutant hab1-1 abi1-2 abi2-2 grown under SDs. Student's t test P value ≤ 0.001 (***) compared to Wild Type.

Supplemental Table S1

Fig 1 A

Genotype	MOCK Rosette leaves	MOCK SE	MOCK range	ABA 25µM Rosette leaves	ABA 25µM SE	ABA 25µM range	t-test MOCK vs ABA 25µM
**WT Col–0**	**10.3**	0.3	9-12	**8.6**	0.6	5-10	**P< 0.001**
aba1–6	**17.8**	0.7	16-22	**11.9**	0.9	8-17	**P< 0.001**
aba2-1	**16.9**	0.7	13-21	**8.8**	0.5	7-13	**P< 0.001**

One-Way ANOVA with post-HOC Tukey HSD Test

MOCK Genotype pair	p-value	inference
**WT Col** vs ***aba1–6***	1.01E-03	** **p<0.01**
**WT Col** vs ***aba2–1***	1.01E-03	** **p<0.01**
aba1–6 vs ***aba2–1***	2.77E-01	**NS**

B

Genotype	Normal Watering Rosette leaves	SE	range	t-test
**WT Col–0**	**13.9**	0.5	12–16	
aba1–6	**17.1**	0.3	16–18	**P<0.001**
ft–10	**40.9**	1.4	36–48	
ft–10 aba1–6	**53.3**	1.3	40–63	**P<0.001**
ft–10 tsf–1	**53.9**	0.6	51–55	
ft–10 tsf–1 aba1–6	**58.4**	1.4	53–64	**P=0.018**
gi–2	**61.9**	1.0	59–67	
gi–2 aba1–6	**62.0**	1.2	54–73	**P=0.945**

C

Genotype	Normal Watering Rosette leaves	SE	range	t-test
**WT Col–0**	**6.5**	0.2	5–8	
aba1–6	**7.8**	0.2	7–9	**P<0.001**
ft–10	**20.8**	0.5	19–23	
ft–10 aba1–6	**24.9**	0.5	24–28	**P<0.001**
co–10	**36.0**	0.7	31–40	
co–10 aba1–6	**36.5**	1.0	28–43	**P=0.484**

Fig 3 A

Genotype	Normal Watering Rosette leaves	SE	range	Low Watering Rosette leaves	SE	range	t-test NW vs LW
**WT Col–0**	**15.9**	0.4	14–18	**11.7**	0.5	9–15	**P<0.001**
cdf1–R cdf2–1 cdf3–1 cdf5–1	**7.8**	0.2	6–9	**5.8**	0.1	5–7	**P<0.001**
cdf1–R cdf2–1 cdf3–1 cdf5–1 gi–100	**9.2**	0.2	8–11	**8.9**	0.2	8–10	**P=0.311**

B

Genotype	Normal Watering Rosette leaves	SE	range	Low Watering Rosette leaves	SE	range	t-test NW vs LW
**WT Col–0**	**55.7**	0.9	51–62	**63.4**	2.4	55–75	**P=0.003**
co–10	**57.5**	1.3	51–65	**73.2**	2.8	54–85	**P<0.001**
cdf1–R cdf2–1 cdf3–1 cdf5–1	**10.5**	0.4	8–14	**10.2**	0.8	7–15	**P=0.729**

G

Genotype	Normal Watering Rosette leaves	SE	range	t-test
**WT Col–0**	**14.0**	0.0	14	
aba1-6	**22.7**	4.0	16-27	**P<0.001**
elf3-1	**4.1**	0.4	4-5	
elf3-1 aba1-6	**6.5**	1.6	4-9	**P<0.001**

Fig 4 A

Genotype	Normal Watering Rosette leaves	SE	range	Low Watering Rosette leaves	SE	range	t-test NW vs LW
**WT Ler**	**8.2**	0.2	7–9	**6.9**	0.1	6–8	**P<0.001**
aba1–1	**9.4**	0.3	8–11	**9.0**	0.3	8–10	**P=0.365**
aba1–3	**9.3**	0.2	8–11	**9.3**	0.2	8–10	**P=0.446**
abi1–1	**8.5**	0.3	7–9	**9.5**	0.3	8–10	**P<0.001**

One-Way ANOVA with post-HOC Tukey HSD Test

Normal Watering Genotype pair	p-value	inference
**WT Ler** vs ***aba1–1***	1.01E-03	** **p<0.01**
**WT Ler** vs ***aba1–3***	3.98E-03	** **p<0.01**
**WT Ler** vs ***abi1–1***	8.56E-01	**NS**
aba1–1 vs ***aba1–3***	9.00E-01	**NS**
aba1–1 vs ***abi1–1***	5.83E-02	**NS**
aba1–3 vs ***abi1–1***	1.30E-01	**NS**

B

Genotype	Normal Watering Rosette leaves	SE	range
**WT Ler**	**37.9**	0.7	33–41
aba1–3	**37.0**	1.2	27–42
abi1–1	**27.0**	1.0	20–32

One-Way ANOVA with post-HOC Tukey HSD Test

Normal Watering Genotype pair	p-value	inference
**WT Ler** vs ***aba1–3***	7.76E-01	**NS**
**WT Ler** vs ***abi1–1***	1.01E-03	** **p<0.01**
aba1–3 vs ***abi1–1***	1.01E-03	** **p<0.01**

Fig 5 F

Genotype	Normal Watering Rosette leaves	SE	range	Low Watering Rosette leaves	SE	range	t-test NW vs LW
**WT Ler**	**35.9**	0.5	32–38	**38.4**	0.8	35–44	**P=0.016**
abi1–1	**27.6**	0.8	23–32	**31.9**	1.3	21–36	**P=0.019**
35S::GI	**13.6**	0.3	12–16	**11.8**	0.2	11–13	**P<0.001**
35S::GI abi1–1	**12.6**	0.5	10–17	**17.5**	0.5	15–20	**P<0.001**

One-Way ANOVA with post-HOC Tukey HSD Test

Normal Watering Genotype pair	p-value	inference
**WT Ler** vs ***abi1–1***	1.01E-03	** **p<0.01**
**WT Ler** vs ***35S::GI***	1.01E-03	** **p<0.01**
**WT Ler** vs ***35S::GI abi1–1***	1.01E-03	** **p<0.01**
abi1–1 vs ***35S::GI***	1.01E-03	** **p<0.01**
abi1–1 vs ***35S::GI abi1–1***	1.01E-03	** **p<0.01**
35S::GI vs ***35S::GI abi1–1***	5.07E-01	**NS**

Fig 6 C

Genotype	Normal Watering Rosette leaves	SE	range
**WT Ler**	**28.9**	1.4	25–37
abi1–1	**17.0**	0.4	16–19
soc1–1	**50.3**	1.9	42–59
abi1–1 soc1–1	**53.7**	1.3	39–61

One-Way ANOVA with post-HOC Tukey HSD Test

Genotype pair	p-value	inference
**WT Ler** vs ***abi1–1***	1.01E-03	** **p<0.01**
**WT Ler** vs ***soc1–1***	1.01E-03	** **p<0.01**
**WT Ler** vs ***abi1–1 soc1–1***	1.01E-03	** **p<0.01**
abi1–1 vs ***soc1–1***	1.01E-03	** **p<0.01**
abi1–1 vs ***abi1–1 soc1–1***	1.01E-03	** **p<0.01**
soc1–1 vs ***abi1–1 soc1–1***	3.25E-01	**NS**

6	D	Genotype	Normal Watering<br>Rosette leaves	SE	range
		**WT Ler**	**8.3**	0.2	8–9
		abi1–1	**8.4**	0.2	8–9
		soc1–1	**12.5**	0.2	12–13
		abi1–1 soc1–1	**15.0**	0.2	14–16

One-Way ANOVA with post-HOC Tukey HSD Test

Genotype pair	p-value	inference
**WT Ler** vs ***abi1–1***	7.96E-01	**NS**
**WT Ler** vs ***soc1–1***	5.69E-06	***** p<0.001**
**WT Ler** vs ***abi1–1 soc1–1***	6.88E-15	***** p<0.001**
abi1–1 vs ***soc1–1***	7.08E-05	***** p<0.001**
abi1–1 vs ***abi1–1 soc1–1***	2.80E-14	***** p<0.001**
soc1–1 vs ***abi1–1 soc1–1***	3.62E-10	***** p<0.001**

**Fig Supp 2**	Genotype	Normal Watering<br>Rosette leaves	SE	range	t-test
	**WT Col–0**	**60.6**	2.1	49-80	
	hab1–1 abi1–2 abi2–2	**80.4**	1.9	68-101	**P<0.001**

Flowering time of mutant and transgenic plants used in this study. Mean values of vegetative leaves and standard error (SE) of plants under different watering conditions. Two tailed Student's *t* test values (*P*) and One-Way ANOVA (*P*) are shown, NS (Not Significant).

Supplemental Table S2

Fig			Tukey HSD	
3	H	GI expression Genotype pair	p-value	inference
		**WT Col–0** vs ***aba1–6***	9.00E-01	**NS**
		**WT Col–0** vs ***elf3–1***	1.01E-03	**** p<0.01**
		**WT Col–0** vs ***elf3–1 aba1–6***	1.01E-03	**** p<0.01**
		**aba1–6** vs ***elf3–1***	1.01E-03	**** p<0.01**
		**aba1–6** vs ***elf3–1 aba1–6***	1.01E-03	**** p<0.01**
		**elf3–1** vs ***elf3–1 aba1–6***	9.00E-01	**NS**
			Tukey HSD	
	I	CO expression Genotype pair	p-value	inference
		**WT Col–0** vs ***aba1–6***	1.29E-01	**NS**
		**WT Col–0** vs ***elf3–1***	8.99E-01	**NS**
		**WT Col–0** vs ***elf3–1 aba1–6***	8.96E-01	**NS**
		**aba1–6** vs ***elf3–1***	3.57E-02	*** p<0.05**
		**aba1–6** vs ***elf3–1 aba1–6***	3.76E-01	**NS**
		**elf3–1** vs ***elf3–1 aba1–6***	5.19E-01	**NS**
			Tukey HSD	
	J	FT expression Genotype pair	p-value	inference
		**WT Col–0** vs ***aba1–6***	9.00E-01	**NS**
		**WT Col–0** vs ***elf3–1***	1.46E-02	*** p<0.05**
		**WT Col–0** vs ***elf3–1 aba1–6***	9.00E-01	**NS**
		**aba1–6** vs ***elf3–1***	6.89E-03	**** p<0.01**
		**aba1–6** vs ***elf3–1 aba1–6***	9.00E-01	**NS**
		**elf3–1** vs ***elf3–1 aba1–6***	1.08E-02	*** p<0.05**
			Tukey HSD	
	K	TSF expression Genotype pair	p-value	inference
		**WT Col–0** vs ***aba1–6***	7.44E-02	**NS**
		**WT Col–0** vs ***elf3–1***	4.35E-01	**NS**
		**WT Col–0** vs ***elf3–1 aba1–6***	1.49E-01	**NS**
		**aba1–6** vs ***elf3–1***	3.44E-03	**** p<0.01**
		**aba1–6** vs ***elf3–1 aba1–6***	9.00E-01	**NS**
		**elf3–1** vs ***elf3–1 aba1–6***	7.37E-03	**** p<0.01**

One-Way ANOVA (*P*) for the expression analysis of Fig 3H-K are shown, NS (Not Significant).

Supplemental Table S3

Allele	Reference	Background
aba1-6	(Niyogi et al., 1998)	Col-0
aba2-1	(Léon-Kloosterziel et al., 1996)	Col-0
ft-10	(Yoo et al., 2005)	Col-0
ft-10 aba1-6	This Work	Col-0
ft-10 tsf-1	(Jang et al., 2009)	Col-0
ft-10 tsf-1 aba-6	This Work	Col-0
gi-2	(Fowler et al., 1999)	Col-1
gi-2 aba1-6	This Work	Col-1
co-10	(Laubinger et al., 2006)	Col-0
co-10 aba1-6	This Work	Col-0
elf3-1	(Zagotta et al., 1992)	Col-0
elf3-1 aba1-6	This Work	Col-0
cdf1-R cdf2-1 cdf3-1 cdf5-1	(Fornara et al., 2009)	Col-0
gi-100 cdf1-R cdf2-1 cdf3-1 cdf5-1	(Fornara et al., 2009)	Col-0
aba1-1	(Koornneef et al. 1982)	L*er*
aba1-3	(Koornneef et al. 1982)	L*er*
abi1-1	(Koornneef et al., 1984)	L*er*
soc1-1	(Onouchi et al., 2000)	L*er*
abi1-1 soc1-1	This Work	L*er*
35S::GI	(Mizoguchi et al., 2005)	L*er*
abi1-1 35S::GI	This Work	L*er*
hab1-1 abi1-2 pp2ca-1	(Rubio et al., 2009)	Col-0
hab1-1 abi1-2 abi2-2	(Rubio et al., 2009)	Col-0

Genotypes used in this study

Fornara F, Panigrahi KCS, Gissot L, Sauerbrunn N, RUhl M, Jarillo JA, Coupland G (2009) Arabidopsis DOF Transcription Factors Act Redundantly to Reduce CONSTANS Expression and Are Essential for a Photoperiodic Flowering Response. Dev Cell **17**: 75–86

Fowler S, Lee K, Onouchi H, Samach A, Richardson K, Morris B, Coupland G, Putterill J (1999) GIGANTEA: a circadian clock-controlled gene that regulates photoperiodic flowering in Arabidopsis and encodes a protein with several possible membrane-spanning domains. EMBO J **18**: 4679–4688

Jang S, Torti S, Coupland G (2009) Genetic and spatial interactions between FT, TSF and SVP during the early stages of floral induction in Arabidopsis. The Plant Journal **60**: 614–625

Koornneef M, Jorna ML, Brinkhorst-van der Swan DLC, Karssen CM The isolation of abscisic acid (ABA) deficient mutants by selection of induced revertants in non-germinating gibberellin sensitive lines of Arabidopsis thaliana (L.) heynh. Theoretical and Applied Genetics **61**: 385–393

Koornneef M, Reuling G, Karssen CM (1984) The isolation and characterization of abscisic acid-insensitive mutants of Arabidopsis thaliana. Physiol Plant **61**: 377–383

Laubinger S, Marchal V, Gentilhomme J, Wenkel S, Adrian J, Jang S, Kulajta C, Braun H, Coupland G, Hoecker U (2006) Arabidopsis SPA proteins regulate photoperiodic flowering and interact with the floral inducer CONSTANS to regulate its stability. Development **133**: 3213–3222

Léon-Kloosterziel KM, Gil MA, Ruijs GJ, Jacobsen SE, Olszewski NE, Schwartz SH, Zeevaart JAD, Koornneef M (1996) Isolation and characterization of abscisic acid-deficient Arabidopsis mutants at two new loci. The Plant Journal **10**: 655–661

Mizoguchi T, Wright L, Fujiwara S, Cremer F, Lee K, Onouchi H, Mouradov A, Fowler S, Kamada H, Putterill J, et al (2005) Distinct roles of GIGANTEA in promoting flowering and regulating circadian rhythms in Arabidopsis. Plant Cell **17**: 2255–2270

Niyogi KK, Grossman AR, Björkman O (1998) Arabidopsis mutants define a central role for the xanthophyll cycle in the regulation of photosynthetic energy conversion. Plant Cell **10**: 1121–1134

Onouchi H, Igeno M, Perilleux C, Graves K, Coupland G (2000) Mutagenesis of plants overexpressing CONSTANS demonstrates novel interactions among Arabidopsis flowering-time genes. Plant Cell **12**: 885–900

Rubio S, Rodrigues A, Saez A, Dizon MB, Galle A, Kim T-H, Santiago J, Flexas J, Schroeder JI, Rodriguez PL (2009) Triple loss of function of protein phosphatases type 2C leads to partial constitutive response to endogenous abscisic acid. Plant Physiol **150**: 1345–1355

Yoo SK, Chung KS, Kim J, Lee JH, Hong SM, Yoo SJ, Yoo SY, Lee JS, Ahn JH (2005) CONSTANS activates SUPPRESSOR OF OVEREXPRESSION OF CONSTANS 1 through FLOWERING LOCUS T to promote flowering in Arabidopsis. Plant Physiol **139**: 770–778

Zagotta MT, Shannon S, Jacobs C, Meeks-Wagner DR (1992) Early-Flowering Mutants of Arabidopsis thaliana. Australian Journal of Plant Physiology **19**: 411–418

Supplemental Table S4

Gene	Forward	Use
aba1–6	GCTCGGAGTAAAGGCGGCGA<br>CAGGAAGTCCCCGTGACGCC	Genotyping
abi1–1	ATGGAGGAAGTATCTCCGGCG<br>TCAGTTCAAGGGTTTGCTCTTGAG	Genotyping
CO WT	atgttgaaacaagagagtaac<br>tcattgtgttactgttatcatctg	Genotyping
co–10	atgttgaaacaagagagtaac<br>gccttttcagaaatggataaatagccttgcttcc	Genotyping
attb pABI1	GGGGACAAGTTTGTACAAAAAAGCAGGCTCTCTTATCCACTTTGCCCGC<br>GGGGACCACTTTGTACAAGAAAGCTGGGTGCGATCGCCGGAGATACTTC	Cloning
ACT	CTCTCCCGCTATGTATGTCGCCA<br>GTGAGACACACCATCACCAG	qPCR
CO	CTACAACGACAATGGTTCCATTAAC<br>CAGGGTCAGGTTGTTGC	qPCR
FT	CTAGCAACCCTCACCTCCGAGAATA<br>CTGCCAAGCTGTCGAAACAATATAA	qPCR
TSF	CTCGGGAATTCATCGTATTG<br>CCCTCTGGCAGTTGAAGTAA	qPCR
SOC1	ATCGAGGAGCTGCAACAGAT<br>GCTACTCTCTTCATCACCTCTTCC	qPCR
GI	AATTCAGCACGCGCCTATTG<br>GTTGCTTCTGCTGCAGGAACTT	qPCR

Primers used in this study
